# Navigating mental health challenges in international university students: adapting to life transitions

**DOI:** 10.3389/fpsyt.2025.1574953

**Published:** 2025-05-05

**Authors:** Soroush Ansari Lari, Maya Salem Zumot, Salim Fredericks

**Affiliations:** ^1^ Royal College of Surgeons in Ireland- Medical University of Bahrain, Muharraq, Bahrain; ^2^ Royal Blackburn Hospital, East Lancashire Teaching Hospitals Trust, Blackburn, United Kingdom; ^3^ Department of Biochemistry, Royal College of Surgeons in Ireland- Medical University of Bahrain, Muharraq, Bahrain

**Keywords:** international students, mental health, life transitions, acculturative stress, coping strategies

## Abstract

Change is an inevitable part of life yet navigating it can be challenging. Students transitioning from school to higher education encounter unique stressors that can significantly impact their mental health. International students face compounded difficulties as they adapt to life abroad, often for the first time, without immediate support from family and friends. This transition introduces independent living, which demands new responsibilities such as managing finances, academics, and personal well-being—tasks that are frequently underestimated. These challenges are exacerbated by cultural adjustments, language barriers, and the isolation that accompanies being far from familiar support systems. Combined, these factors contribute to heightened stress levels and increased risk of mental health concerns in this population. However, the ability to build resilience and adopt effective coping mechanisms plays a crucial role in mitigating these challenges. Among these, social support and culturally tailored university programs consistently emerge as the most effective for enhancing resilience. Further strategies such as developing strong social networks, practicing self-care, and seeking institutional support can enhance students’ ability to manage stress and adapt to their new environment. Additionally, fostering cultural competence, promoting mental health awareness, and providing tailored resources for international students can further bolster their mental well-being. Effective coping strategies identified in the literature include social support, self-compassion, culturally sensitive university programs, and mental health literacy initiatives. This paper explores the unique mental health challenges faced by international university students and highlights coping strategies aimed at promoting resilience and improving students’ capacity to thrive during this pivotal life transition.

## 1 Introduction

Life transitions are periods of significant change that require individuals to adapt, develop new skills, and navigate uncertainty (1, 2). One of the most challenging transitions occurs when individuals enter university, as they take on new academic, social, and personal responsibilities (1, 3). University life introduces unique stressors, including large-group teaching environments that may hinder social connections (4) and higher academic demands that can cause students to question their abilities (5).

For international students, these stressors are often intensified (6–8). Many are living independently for the first time, far from familiar support systems. They may struggle with homesickness, loneliness, and cultural barriers such as language difficulties and unfamiliar teaching styles (6–8). These obstacles are often more severe for non-English-speaking students in English-speaking countries, where limited language proficiency can lead to alienation and difficulty forming social connections (9, 10). Additionally, international students often face significant financial pressures, balancing high tuition fees and living expenses (11, 12).

To better understand these experiences, Berry’s Acculturative Stress Model provides a useful framework for examining how international students navigate cultural transitions (13). The model highlights how stress arises when individuals adapt to new cultural and social environments, particularly when facing language barriers, discrimination, and cultural isolation (7, 10). Importantly, it emphasizes the role of personal coping strategies and social support in reducing acculturative stress, making it highly relevant to understanding the mental health challenges and resilience-building efforts of international students (10). However, these experiences are not uniformly experienced by all students alike; factors such as transnational identity and gender shape the way individuals experience and respond to stressors (10). Gender roles and cultural norms affect the type of coping strategies used, the availability of social support, and susceptibility to stress. Additionally, transnational identity, where students navigate multiple cultural affiliations, can make the adaptation process even more complex (14). The principles of this framework will serve as a foundation throughout this paper to critically analyze how resilience and adaptive coping strategies can mitigate the mental health impact of acculturative stress during university transitions.

Stress itself is a double-edged sword—its effects depend on how individuals perceive their situation and the coping resources they can access (15). While adaptive strategies can enhance academic performance, overwhelming stress may lead to depression and anxiety (16, 17).

As the number of international students grows (18), issues like culture shock and adjustment challenges increasingly affect mental health. Research using semi-structured interviews has found that students who use positive coping strategies—such as seeking social support, engaging in exercise, and maintaining positive thinking—are more likely to overcome depression. Conversely, those struggling with language barriers and social isolation continue to face significant challenges, underscoring the need for effective support systems and accessible mental health resources (19).

This review identified studies through PubMed, Scopus, and Google Scholar using keywords such as ‘international university students,’ ‘mental health,’ ‘coping strategies,’ and ‘resilience.’ It included peer-reviewed empirical research with validated measurement tools on psychological well-being, adaptation, and protective factors.

This mini review aims to explore the unique mental health challenges faced by international students and evaluate the coping strategies most effective in fostering resilience.

## 2 Mental health as an international student

International students face unique and complex challenges that place them at heightened risk for mental health issues. A growing body of research has explored the prevalence of depression, anxiety, and psychological distress among this population, and the underlying factors contributing to their vulnerability. However, existing literature is often fragmented, limited geographically, and focuses predominantly on deficits rather than resilience.

Maharaj et al. (2024) conducted a systematic review of 19 studies involving 7,247 participants, focusing on the mental health of international students in Australia. The review highlights widespread mental health challenges, including elevated rates of anxiety (2.4–43%), depression (3.6–38.3%), and psychological distress (31.6–54%), along with high levels of loneliness (60–65%), financial strain (15.4–95%), and discrimination (9–50%) (20). A major strength lies in the review’s comprehensive scope, synthesizing data to reveal the multifactorial nature of international students’ struggles. However, the review is limited by its geographical concentration on Australia, raising questions about the global applicability of its findings. Furthermore, reliance on predominantly cross-sectional and self-reported data weakens causal inferences and introduces potential biases, while variations in tools and measures between studies hinder comparability. Notably, the review exposes critical service gaps, highlighting underutilization of mental health services due to stigma, low mental health literacy, and cultural barriers — yet it does not deeply explore potential facilitators of help-seeking, an area warranting further investigation. Future research should prioritize longitudinal designs and standardized measures to strengthen the evidence base and expand analyses to non-Australian contexts.

Kabir et al. (2021) extend the conversation by examining a rarely studied population of 200 non-native international dental students in Bangladesh. They report strikingly high rates of depression (51.5%) driven by financial strain (77.3%), homesickness (80.5%), language barriers (60%), and poor living conditions (79.7%) (21). This study’s value lies in drawing attention to international students in non-Western, non-English-speaking contexts — a notable contrast to Australian or Western-centric studies. The use of a validated scale (CES-D-10) strengthens its methodological rigor. However, the small, convenience-based sample limits generalizability, and its cross-sectional design cannot establish causality. Furthermore, as the study was conducted during the COVID-19 pandemic, it is difficult to distinguish pandemic-specific stressors from pre-pandemic challenges, limiting its applicability to post-pandemic realities. Despite these limitations, the study highlights the exacerbated mental health burden among students navigating both academic demands and cultural isolation in less globally dominant host nations — a perspective underexplored in existing literature.

Adding an international comparative dimension, Alnaim et al. (2024) and Hosseinpur et al. (2023) provide cross-sectional data on international students in the U.S., UK, and Australia, and the UK, respectively. Alnaim et al.’s study of 401 Saudi students found a high prevalence of depression (40.4%), linked to prior mental illness and feelings of alienation (22), while Hosseinpur et al. found a slightly lower rate (34.6%) among 153 postgraduate students, driven by financial hardship, homesickness, and communication barriers (23). Both studies are strengthened by their use of validated tools (PHQ-8 and CES-D-10) and highlight critical underuse of mental health services, with Hosseinpur et al. noting only 13.1% of participants sought help. However, the overrepresentation of one nationality (Saudis) in Alnaim et al. and focus on a single institution in Hosseinpur et al. limits broader applicability. Moreover, their reliance on self-reports introduces bias, and the absence of qualitative exploration limits understanding of nuanced cultural dynamics. Importantly, these studies demonstrate consistent patterns of depression and service avoidance, regardless of country, signaling a universal need for culturally attuned interventions, yet they lack an in-depth analysis of resilience factors that may mitigate these challenges.

Yeung et al. (2021) offer a broader U.S. perspective, analyzing 44,851 college students, including 2,423 international students, through the American College Health Association-National College Health Assessment (ACHA-NCHA) survey. Contrary to vulnerability-centric narratives, international students were less likely to report anxiety or dual depression-anxiety diagnoses compared to domestic peers, yet reported higher rates of depressive symptoms (42.4%) and suicide attempts (2.2%) (24). This paradox suggests that while international students may exhibit outward resilience, they remain vulnerable to severe untreated symptoms — potentially masked by stigma and help-seeking hesitancy. This study is strengthened by its large sample size and use of a validated survey tool, but as a cross-sectional study, it cannot establish causality. Furthermore, it relies solely on self-reported data, which may be subject to social desirability bias and underreporting due to stigma. Although it highlights a clear discrepancy between reported symptoms and formal diagnoses, the study lacks deeper subgroup analysis of international students, such as differences by country of origin or cultural background, limiting its ability to identify specific at-risk populations. The absence of qualitative data also prevents insight into why students may avoid diagnosis or treatment, which is crucial for developing targeted interventions.

Prasath et al. (2022) introduce a resilience-focused perspective, investigating how psychological capital (PsyCap) including hope, self-efficacy, resilience, and optimism mediates and moderates psychological distress among 188 international students in the U.S. during COVID-19 (25). The finding that well-being buffers against distress is a valuable addition to the discourse, emphasizing not just vulnerability but also protective factors. However, the study’s small, regionally limited sample and reliance on self-report measures raise concerns about representativeness and possible response bias. The pandemic-specific context further limits generalizability to post-pandemic experiences. Moreover, while the use of the PERMA model adds conceptual richness, the cross-sectional design prevents any causal claims, and the study does not explore how structural factors (e.g., visa stress, discrimination) may interact with PsyCap. Nevertheless, this study is critical in shifting the focus from deficits to strengths, suggesting that interventions enhancing PsyCap may mitigate psychological stressors; a theme underexplored in the largely deficit-focused literature.

De Moissac et al. (2020) provide a Canadian perspective, surveying 932 students (21% international) across two universities. Interestingly, international students reported better overall mental health, higher life satisfaction, and self-esteem compared to domestic peers — though they were less likely to seek help (26). This contradicts much of the literature highlighting distress among international students, pointing instead to possible protective cultural or personal resources. Strengths include a large sample, bilingual context (English/French), and detailed psychological measures. Yet, the very small international sample at one institution (n=38) limits subgroup analysis, and, like other studies, self-report measures may obscure underlying issues due to stigma. The study’s unexpected findings highlight the need for more nuanced, culturally informed research into what promotes flourishing among international students — a dimension largely neglected in deficit-centered research.

Jin et al. (2024) examined how beliefs about living conditions, emigration intent, and fate influence depression in 1,014 Chinese international student returnees. Using a WeChat-recruited cross-sectional survey and PHQ-9 assessments, they found that students who believed they could change their environment had lower depression, while those with stronger emigration intent also reported less depression. In contrast, stronger belief in fate was linked to higher depression (27). The study’s strengths include its large sample size, use of a validated depression measure, and Bayesian analysis, which enhances statistical accuracy. However, its reliance on self-reported data introduces bias, and the cross-sectional design limits causal inferences. Additionally, recruiting participants through WeChat may exclude less tech-savvy individuals, reducing sample representativeness. While the study provides insight into mental health challenges among returnees, it does not deeply explore factors like access to mental health services or sociocultural support, both crucial for understanding international student well-being. Future research should employ longitudinal designs to assess mental health trajectories over time and consider qualitative methods for a deeper understanding of acculturative stress and resilience.

All-in-all, these studies highlight mental health challenges among international students, consistently linked to acculturative stress, financial strain, and service underuse. However, they also reveal contradictory findings on vulnerability versus resilience, underscoring a need to move beyond one-dimensional portrayals. Methodological limitations are pervasive, including small, convenience samples, cross-sectional designs, and self-reported data which reduces the strength of causal claims. With fewer robust studies from non-English-speaking countries, the global applicability of findings is also narrowed. Future research should prioritize longitudinal, multi-country studies employing mixed methods to capture both distress and resilience. Crucially, interventions should not only address vulnerabilities but also harness protective factors like PsyCap, building culturally responsive support systems that reflect international students’ complex realities.

## 3 Adapting to life changes

When moving to a different country, it is expected that one’s routine and overall life will change significantly (28). These adjustments can lead to acculturative stress, making coping strategies essential for international students’ mental well-being. Among these, social support has been identified as one of the most effective protective factors. Kristiana et al’s meta-analysis of 8 studies found that overall a social support system plays a significant role in reducing acculturative stress among international students. The study, which examined data from students of various cultural backgrounds, highlighted that individuals from collectivist cultures tend to benefit more from social support than those from individualistic backgrounds. However, the impact of social support is influenced by multiple factors, such as length of stay and type of support received. The study found that overall many factors affect how much social support can impact one’s stress, such factors include length of stay and type of support being received. Despite its benefits, social support is not the only coping mechanism available to international students; other strategies, such as self-compassion, cognitive reframing, and structured university interventions, also play critical roles in mental health outcomes. Research suggests that self-compassion may be particularly beneficial for students from individualistic cultures, while university-led initiatives—such as peer mentoring programs, culturally tailored counseling services, and mental health literacy workshops—provide institutional support that enhances well-being and facilitates adjustment, especially in the early stages of transition (18). Strengths of this paper include the comprehensive meta-analysis as it looked at papers from 2009–2019 in different regions of the world, and empirical validation as it uses statistical analyses (PRISMA framework, heterogeneity tests, and moderator analysis) to validate findings. However, some limitations include high heterogeneity in effect sizes that makes generalization difficult, while the lack of consideration for factors like marital status and personality traits limits the scope of analysis. Publication bias and missing studies may distort the findings, and reliance on cross-sectional data hinders long-term trend analysis. Additionally, the use of varied measurement tools for social support and acculturative stress reduces consistency across studies.

Another important factor in student adaptation is lifestyle satisfaction. A study by Machul et al. compared lifestyle satisfaction between Polish and international students, finding that although international students scored lower on lifestyle measures, their overall satisfaction with life was comparable to that of locals. This suggests that despite the additional challenges they face, international students demonstrate resilience when adapting to a new culture (29). One important limitation is that the study did not assess cultural adaptation directly, which could influence stress and lifestyle differences influencing the results. In addition, the sample was drawn from a single medical university in Poland, limiting generalizability. Furthermore, there was a gender imbalance, with more female participants in the Polish group, potentially affecting the results as differences between males and females were also not directly studied. However, the study is reliable as it used comparative analysis comparing internationals to the locals and used validated tools, FLQ, SWLS, PSS-10, to measure lifestyle practices and satisfaction.

Adaptation challenges do not end upon returning home, as international students may also experience reverse culture shock (30). A study conducted in Pakistan by Akhtar et al. found that reverse culture shock negatively correlates with psychological well-being and the length of stay abroad does not affect the relationship. Overall, it was found that younger returnees are more likely to be impacted negatively and that 29.3% of participants experienced mild to severe depression while 54.1% reported moderate to severe anxiety (31). These findings underscore the need for continued mental health support not only during students’ time abroad but also during their reintegration process. Universities and policymakers should consider extending transitional support services to assist students in readjusting to life in their home countries. An important strength of this study is the use of validated scales like the Reverse Culture Shock Scale, Beck Anxiety Inventory, and WHO-5 Well-being Index for data collection. Some limitations include the small sample size which was predominantly male, limiting the generalizability of findings. Its cross-sectional design prevents insights into the long-term effects of reverse culture shock. Additionally, reliance on self-reported data introduces potential bias, and the study focuses solely on Pakistani returnees, making it less applicable to other cultural contexts.

Another study done by Jin et al. (2024) looked at 20 Chinese international students returning post-COVID-19 found that people’s personality types significantly influenced their experience when re-integrating into their home country. It categorized the students into homestayers, wayfarers, and navigators and identified how each group of students needed different levels of support reinforcing the need for comprehensive support systems and policies should address the varying levels of support needed by different student groups, ensuring identity continuity while fostering an inclusive and diverse society (32). The study’s strength lies in its nuanced qualitative approach, which captures the complexity of returnees’ experiences beyond simplistic adjustment models. However, its small, homogenous sample limits generalizability, and reliance on self-reported narratives introduces potential bias. The absence of direct mental health assessments weakens the link between identity conflict and psychological distress. Additionally, the study does not account for external factors like government policies or economic conditions that may influence reintegration. Despite these limitations, it provides valuable insights into the intersection of identity, culture, and mental well-being, emphasizing the need for targeted support systems for returning students facing cultural alienation. The study suggests that unresolved identity struggles can manifest in long-term emotional distress, highlighting the importance of mental health interventions tailored to the unique challenges faced by international student returnees.

## 4 Discussion

Mental health plays a crucial role in student life, particularly for international students who face additional stressors such as acculturative stress, social isolation, financial strain, anxiety, depression, and language barriers while striving to achieve academic success. However, responses to these challenges vary. Some students demonstrate resilience and adapt effectively, while others experience significant distress, requiring targeted cultural and psychological support. This contrast highlights the need for individualized mental health interventions that acknowledge both resilience and vulnerability.

This review highlights the importance of personalized mental health support, advocating for tailored programs considering diverse cultural backgrounds and individual coping styles. Social support has been identified as a key factor in successful adaptation, benefiting students across all cultural contexts. However, its impact is not uniform—students from collectivist backgrounds often derive greater benefits from communal support whereas those from individualistic cultures may rely more on personal coping strategies such as cognitive reframing or self-compassion. These mixed findings suggest that interventions should incorporate both social and individual approaches to effectively support students from different backgrounds meaning they have different needs.

Several studies included in this review exhibit inherent biases. Cross-sectional designs limit causal interpretations, and self-reported data are prone to social desirability bias. Cultural factors may also influence help-seeking behavior, potentially leading to underreporting of mental health symptoms. Acknowledging these biases is essential for interpreting the findings accurately.

Despite the availability of mental health resources, many students hesitate to seek help due to cultural stigma, lack of awareness, or discomfort in reaching out. Addressing these barriers requires universities to actively promote mental health awareness, normalize discussions around well-being, and foster environments where students feel safe accessing support services.

Some limitations of this mini-review include the type of studies included as most were cross-sectional limiting sights into long-term mental health trajectories. Additionally, few studies explore gender-based differences or the influence of transnational identity, leaving gaps in understanding how these variables shape mental health outcomes. Future research should prioritize longitudinal, mixed-method studies and explore demographic subgroups to provide a more nuanced understanding. Expanding on research in this field will help refine existing support systems and ensure they effectively meet the diverse needs of student populations.

Findings suggest that while multiple coping strategies are beneficial, social support networks and culturally sensitive university interventions consistently show the highest efficacy. Universities should prioritize peer mentoring programs, tailored counseling services, and mental health literacy workshops to better support international students.

By strengthening coping mechanisms and reducing barriers to care, universities can equip students with essential skills that extend beyond their academic journey, promoting long-term well-being and success.
